# Quality of Life after Brain Injury in children aged six and seven years (QOLIBRI-KIDDY) – development and scale analysis of the first disease-specific self-report instrument for young children after traumatic brain injury

**DOI:** 10.1186/s41687-025-00890-5

**Published:** 2025-05-14

**Authors:** Nicole von Steinbuechel, Marina Zeldovich, Fabian Bockhop, Ugne Krenz, Dagmar Timmermann, Anna Buchheim, Inga K. Koerte, Michaela Veronika Bonfert, Steffen Berweck, Matthias Kieslich, Knut Brockmann, Maike Roediger, Sven Greving, Axel Neu, Ulrike Wartemann, Joachim Suss, Christian Auer, Holger Muehlan, Katrin Cunitz

**Affiliations:** 1 Institute of Psychology, University of Innsbruck, Universitaetsstr. 5-7, Innsbruck, 6020 Austria; 2 Faculty of Psychotherapy Science, Sigmund Freud University Vienna, Vienna, Austria; 3 Hospital for Forensic Psychiatry and Psychotherapy Goettingen, Correctional Center Lower Saxony, Ulrich-Venzlaff-Strasse, 237081 Goettingen, Germany; 4 University Medical Center Goettingen, Robert-Koch-Str. 40, 37075 Goettingen, Germany; 5 Department of Psychosomatic Medicine and Psychotherapy, Division Medical Psychology and Medical Sociology, University Medical Center Goettingen, Waldweg 37a, 37075 Goettingen, Germany; 6 cBRAIN/Department of Child and Adolescent Psychiatry, Psychosomatics, and Psychotherapy, LMU University Hospital, Ludwig-Maximilian University, Nussbaumstrasse 5, 80336 Munich, Germany; 7 Department of Pediatric Neurology and Developmental Medicine and LMU Center for Development and Children With Medical Complexity, Dr. Von Hauner Children’s Hospital, LMU University Hospital, Lindwurmstr. 4, 80337 Munich, Germany; 8 Specialist Center for Paediatric Neurology, Neurorehabilitation and Epileptology, Schoen Klinik, Krankenhausstraße 20, 83569 Vogtareuth, Germany; 9 Department of Paediatric Neurology, Hospital of Goethe University, Theodor-Stern-Kai 7, 60590 Frankfurt am Main, Germany; 10 Interdisciplinary Pediatric Center for Children with Developmental Disabilities and Severe Chronic Disorders, Department of Pediatrics and Adolescent Medicine, University Medical Center Goettingen, Robert-Koch-Str. 40, 37075 Goettingen, Germany; 11 Pediatric Cardiology, University Hospital Muenster, Albert-Schweitzer-Campus 1, 48149 Muenster, Germany; 12 Department of Neurology and Neuropediatry, VAMED Klinik Geesthacht GmbH, Johannes-Ritter-Straße 100, 21502 Geesthacht, Germany; 13 Department of Neuropediatrics, VAMED Klinik Hohenstuecken GmbH, Brahmsstraße 38, 14772 Brandenburg an der Havel, Germany; 14 Department of Pediatric Surgery, Wilhelmstift Catholic Children’s Hospital, Liliencronstraße 130, 22149 Hamburg, Germany; 15 Department of Neurosurgery, Johannes Kepler University Linz/Kepler University Hospital GmbH, Altenberger Straße 69/Wagner-Jauregg-Weg 15, Linz, 4020 Austria; 16 Department of Health and Prevention, University of Greifswald, Robert-Blum-Str. 13, 17487 Greifswald, Germany; 17 Medical Department, HMU Health and Medical University Erfurt, Anger 66, 99984 Erfurt, Germany

**Keywords:** Health-related quality of life, Traumatic brain injury, Young children, Patient-reported outcomes

## Abstract

To date, there are no age-appropriate instruments for assessing the subjective impact of pediatric traumatic brain injury (TBI) sequelae on multiple domains of health-related quality of life (HRQoL) in young children. The present study therefore aims to develop and examine the psychometric properties of a new disease-specific, self-reported HRQoL instrument, the Quality of Life after Brain Injury for children aged 6–7 years (QOLIBRI-KIDDY). Questionnaire development included focus group interviews, cognitive debriefings, and Delphi expert panels. The pilot version of the instrument was tested in 72 children (6.00–7.92 years of age; 60% boys; 86% after mild TBI). After item reduction based on a confirmatory scale analysis considering the six-factor structure of the questionnaire versions for older children, adolescents, and adults (Cognition, Self, Daily Life & Autonomy, Social Relationships, Emotions, Physical Problems), its reliability and validity were investigated. The final version of the QOLIBRI-KIDDY comprises 23 items. Psychometric analyses indicated internal consistency to be satisfactory (*ɑ* = 0.49–0.72; *ω* = 0.57–0.78). Construct validity suggested the expected overlap between generic HRQoL and TBI-specific HRQoL (*r* = 0.17–0.36). There were small (*r* > 0.2) to moderate (*r* > 0.3) correlations between lower TBI-specific HRQoL and participants with lower learning rates, anxiety, depression, and post-concussion symptoms, particularly on the Cognition, Social Relationships, Emotions, and Physical Problems scales. The comparison of known groups revealed significant moderate and significant effects for lower HRQoL in children with depressive symptoms on the Emotions scale (*d* = − 0.46) and with post-concussion symptoms on the Cognition (*d* = − 0.42) and Social Relationships scales (*d* = − 0.56). The QOLIBRI-KIDDY is a comprehensive, yet economical tool, comparable in content and items to the other age-adapted QOLIBRI versions. Its application has the potential to provide longitudinal data on subjects after TBI from childhood to older age, with a subjective perspective that can contribute to improving the therapy, rehabilitation, and daily life of young children.

## Introduction

Pediatric traumatic brain injury (TBI) is one of the most common causes of acquired disability in children and adolescents [1]. In Germany, an incidence rate of 687/100,000 in children up to 18 years [2] has been reported. The present study is informed particularly by the incidence rates for children aged six to seven years. The hospitalization rates in this age group are comparable with the relatively lower rates in older children [6 years: 547/100,000; 7 years: 530/100.000; 2].

Pediatric TBI negatively affects different life domains such as physical well-being [3], social functioning [4], self-perception [5], cognition [6], and mental health [7]. All of these dimensions and their impairment are relevant to pediatric health-related quality of life [HRQoL; 8]. Examining the effects of pediatric TBI on outcomes in children between the ages of five and eight years, outcomes such as inhibitory control seem to be more vulnerable to the impact of TBI at this age than at later ages [9]. A study by Keenan and colleagues [10] in preschool (aged 2.5 years) and school children (aged 6–11 years) after TBI has shown that younger children are at a higher risk of decreased emotional control and increased affective and behavioral disturbances. This means that the impact of TBI on HRQoL is notable in young children [11]. Follow-up studies have shown that HRQoL is significantly worse in individuals with early TBI compared to healthy controls, even 15 years after the injury [12]. These individuals also experienced more severe depressive symptoms, reduced cognitive flexibility, and greater difficulty with their social communication skills [12]. Overall, evidence from TBI studies suggests that HRQoL is reduced in girls [13], older children [14], and in those after moderate to severe TBI [15]. Importantly, HRQoL after TBI is associated with children’s functional recovery [16], underlining the importance of recovery processes. Poorer HRQoL is often observed in connection with commonly reported post-concussion symptoms, such as cognitive [e.g., fatigue; 17], sensory [e.g., light sensitivity; 14], or physical [e.g., sleep problems; 18] difficulties. In addition, poor HRQoL after pediatric TBI is associated with psychiatric symptoms, such as higher levels of depression [19] and anxiety [20]. Hence, comprehensive physical and psychological interventions should be made available to individuals after pediatric TBI based on comprehensive diagnostic procedures. In fact, Varni and colleagues [21] suggest that validated evaluations of TBI-related outcomes should also include the subjective perspective of health problems and should cover various domains of life.

In recent decades, the diagnostic process in research and healthcare has seen an increased focus on assessing multiple outcomes after TBI from a more patient-centered perspective using patient-reported outcome measures [PROMs; 22, 23]. PROMs are standardized and validated questionnaires that capture information concerning patients’ views with respect to their health and HRQoL [24, 25]. HRQoL-related PROMs can be categorized as either generic or disease-specific. Generic tools measure HRQoL across a wide range of medical conditions, allowing a general assessment of patients’ subjective views on health, care status, and the effectiveness of interventions [26]. In contrast, disease-specific instruments capture the specific and individual impact of a particular health condition (e.g., TBI) on HRQoL. In fact, disease-specific assessment is preferred because it is more sensitive to the relevant problem areas of TBI patients [26].

The growing awareness of the importance of integrating children’s health perspectives into clinical decisions has led to the development of multiple generic HRQoL instruments for pediatric populations [27, 28]. A well-established and psychometrically sound instrument for assessing disease-specific HRQoL after TBI is the Quality of Life after Brain Injury [QOLIBRI-Adult; 29], which has recently also been developed and validated for use in children and adolescents aged 8–17 years [QOLIBRI-KID/ADO; 30]. In fact, most research on children’s HRQoL has focused on children over the age of eight [31–33]. However, existing literature shows that children aged six [34] or five years [35, 36] are clearly able to report on their health and satisfaction with different life aspects. However, to date, no TBI-specific HRQoL instrument has been developed specifically for young children (i.e., seven years of age and younger).

The World Health Organization (WHO) has issued guidelines for the development of HRQoL instruments for pediatric populations, which require that such measures should be age-appropriate and child-centered. They should preferably take into account self-reporting, be usable independently of the health status and across different cultures, and should include both positive and negative aspects [37]. Accordingly, the aim of the current study is the development and initial validation of a QOLIBRI-KIDDY self-report version for six- and seven-year-old children after pediatric TBI.

## Materials and methods

### Participants

Participants (*N* = 72) were recruited as part of the QOLIBRI-KIDDY/KID/ADO study, which was conducted in Germany from January 2019 to February 2023. In developing the QOLIBRI-KIDDY version, the inclusion criteria were: age from 6–7 years; diagnosis of TBI (at least three months until seven years ago); information on TBI severity (either based on the Glasgow Coma Scale [GCS; 38] score at the time of the TBI, the International Statistical Classification of Diseases and Related Health Problems [ICD-10; 39] code, or on the clinical description); outpatient status or beginning of hospital discharge; and ability to understand and respond to the questions. Patients were excluded if they were in a vegetative state, had suffered a spinal cord injury, or displayed severe mental disease or epilepsy before TBI. Furthermore, children with diseases leading to death (e.g., severe tumor or heart disease) and those who had sustained a very severe poly-trauma were not eligible to participate.

Medical consecutive records from 15 German hospital registries were screened to extract diagnoses and to determine eligibility based on the pre-specified inclusion and exclusion criteria listed above. Following this process, invitations were sent to potential participants by the recruiting clinic staff. Families received detailed information about the purpose and procedures of the study before signing the informed consent form. Written informed consent for study participation was obtained from the parents and the children.

### Ethical approval

The Ethics Committee of the University Medical Center Goettingen approved the study (application no.: 19/4/18). In accordance with the requirements of the International Committee of Medical Journal Editors (ICMJE), the study was registered within the German Register of Clinical Studies (DRKS; https://drks.de/search/de/trial/DRKS00032854), which works closely with the WHO.

### Procedure

Data were assessed analogously to the data collection for older children through interviews with children, parental reports and medical records [30]. Face-to-face interviews with children were conducted either online, at the recruiting hospitals, or at home. The children were interviewed about their HRQoL by trained, supervised medical or psychological staff. The staff members were responsible for explaining and practicing the tasks and reading the questions to the children, who were presented with the smiley response options on screen or in print. The children were then asked to provide their answers verbally or by pointing to the corresponding option. Additionally, neuropsychological screening tests were performed. Parents completed the questionnaires at the recruiting hospitals or sent them back by mail. The following variables were obtained from parental reports: gender of children and parents, family situation, educational facility, educational attainment as well as the proxy-reported mental health of their children. We aggregated “physical, sensory, and cognitive problems” after TBI (smell, taste, vision, hearing, learning problems, seizures, difficulties with language/speech, back pain, difficulties moving hands/arms, walking problems) to form a summary variable. Data from the medical records included the age of the children, TBI severity (mild, moderate, severe), time since injury, and presence of cerebral lesions.

### Item pool generation and selection

The methodology employed to create the item pool for the newly developed TBI-specific HRQoL questionnaire for children and adolescents aged 8 to 17 years is described in detail in a separate publication [30]. This procedure was also followed during the development of the KIDDY version: to generate the items, several focus group interviews were performed with children after TBI aged 5 to 17 years and with their parents [40]. The main finding of these problem-oriented focus group interviews was that five-year-old children are already able to discuss and report on their HRQoL. In contrast to their parents and older children, children between the ages of five and seven years focused more on cognition and self-related issues than on aspects of autonomy.

Further items were added to the pool based on a literature review on QoL, HRQoL, and TBI-related instruments. Additionally to our team, national and international pediatric experts supported the development of the item pool (see acknowledgements). Predominantly, they were pediatric neurologists and psychologists, specializing in pediatric TBI, mental health and well-being, neuropsychological rehabilitation and Patient-Reported Outcome development. Items were then translated from German into English according to accepted guidelines [41, 42]. In the next step, five national and international experts rated the items based on relevance of content, understandability, and appropriateness of item wording. This resulted in either the inclusion or exclusion of the items. Two Delphi panels of experts then rated the items according to criteria such as relevance, clarity, and age appropriateness. Items that received at least 66% agreement from the panels were retained.

Finally, during the cognitive debriefing of the instructions and items of the preliminary German questionnaire, the children were asked if they had difficulties understanding the sentences/questions, what they meant to them, how they would rephrase them, if they were not clear (e.g., the German word “KiTa” (“kindergarden”) was not clear to every child, so we changed it to the German word “Kindergarten”). They were also asked whether the question was relevant to their situation and whether the response options were clear and consistent with the question. The analyses of the cognitive debriefing results showed that with very young children, the examiner needs to pay special attention to ensure that the child is consistently aware of the temporal reference contained in the questions (now and in the past week). Providing a validated smiley response scale [43] as a visual response option was found to be very useful, and examples helped the young children to understand the questions. Therefore, the final version of the questionnaires used in the current study included smileys and illustrative examples.”

For the KIDDY version, a total of 57 items were aggregated and administered to the study participants. In order to be able to use the questionnaire with both preschool and school-aged children, some items differed in their wording, referring to HRQoL aspects in preschool (i.e., kindergarten) and school, respectively. The final item selection was based on the comparability with the adult QOLIBRI and the QOLIBRI-KID/ADO versions, content and test length appropriate for the age group, and psychometric characteristics (e.g., reliability of the scales using Cronbach’s α and McDonald’s ω). As a measure of the validity of the scales, we examined how well a factor reproduced the covariance matrix.

### PROMs

The *QOLIBRI-KIDDY* questionnaire was developed as a self-report for children after TBI and as a proxy-report measure for their parents. It is recommended that the proxy-reports only be used when the individuals concerned are not capable of understanding and/or answering the questions. This study focused on the findings from the children’s self-reports. Satisfaction concerning Cognition, Self, Daily Life and Autonomy, and Social Relationships is rated on a five-point Likert-type scale (not at all (1); slightly (2); moderately (3); quite (4); very (5)) regarding the present and the past week (“How satisfied are you with…?”). Feeling bothered is rated for Emotions and Physical Problems (“How bothered are you by…?”). Verbal responses are complemented by a validated visual smiley scale for better understanding [43]. For scoring, the latter two scales are re-coded to match the positively worded scales. Scale scores are calculated as the average of the respective items and are linearly transformed to a 0–100 scale, with higher scores indicating better TBI-specific HRQoL. The total score can be calculated as the average of the six scales.

Generic HRQoL was assessed by means of the *Young Child Report* (ages 5–7 years) of the *Pediatric Quality of Life Inventory 4.0* [PedsQL^TM^; 44] using the generic core scales with 23 items. The children self-reported potential problems occurring during the past four weeks on a three-point Likert-type scale (not at all (0); sometimes (2), a lot (4)). Items are reverse scored and linearly transformed to a 0–100 scale, with higher scores indicating better generic HRQoL. Four domains of HRQoL (physical, emotional, social, and school functioning) can be summarized as two total scores: a Physical Health Summary Score (eight items) and a Psychosocial Health Summary Score (15 items).

The *Generalized Anxiety Disorder 7-Item Scale* (GAD-7; [45]) is a screening instrument for generalized anxiety based on the Diagnostic and Statistical Manual of Mental Disorders (DSM-IV; [46]). It measures seven symptoms that may have bothered children in the past two weeks, as reported by parents on a four-point Likert-type scale (not at all (0); several days (1); more than half the days (2); nearly every day (3)). The summarized total score indicates minimal (1–4), mild (5–9), moderate (10–14), or severe symptoms (15–21).

The *Patient Health Questionnaire 9-Item Scale* (PHQ-9; [47]) is a screening tool for major depression based on the DSM-IV criteria [46]. Parents rate nine problems that may have bothered children over the past two weeks on a four-point Likert-type scale (not at all (0); several days (1); more than half the days (2); nearly every day (3)). The summarized total score indicates minimal (1–4), mild (5–9), moderate (10–14), and severe symptoms (15–27).

The *Post-Concussion Symptom Inventory for ages 5–7* (PCSI-SR5; [48]) was translated into German and linguistically validated by our research group; it includes five different physical, cognitive, emotional, and attentional symptoms before and after TBI. The PCSI-SR5 is answered on a three-point Guttman scale (no (0); a little (1); a lot (2)). In the present study, only current symptoms (yesterday and today) are assessed by self-report. The total score is categorized as being above, below, or within the average range for the age group (M ± 1 SD).

The investigators assessed the functional recovery of the children at the time of the study using the *King’s Outcome Scale for Closed Head Injury* (KOSCHI; [49]). Those in a vegetative state were excluded based on the eligibility criteria. The rating started with lower/upper severe disability (3a/b), progressed to lower/upper moderate disability (4a/b), and good/full recovery (5a/b).

The *Rey Auditory Verbal Learning Test* (RAVLT [50, 51]; was used to determine the ability to remember and learn verbal information. Participants are required to reproduce fifteen words presented aloud by examiners in eight trials. The learning rate is calculated by subtracting the number of words correctly recalled in trial I from those recalled in trial V. It was categorized as above, below, or within the sample mean (*M* ± 1*SD*).

### Statistical analyses

In order to allow the characteristics of the two age-adapted versions of the questionnaire to be compared directly, the psychometric testing of the QOLIBRI-KIDDY followed the scheme used in analyzing the QOLIBRI-KID/ADO [30]. Prior to data analyses, missing values in the QOLIBRI-KIDDY, GAD-7, PHQ-9, and PCSI-SR5 were replaced with scale means to avoid data loss if less than one third of the responses per scale was missing.

Descriptive statistics were provided for sociodemographic (children and parents), injury-related sample characteristics, and response patterns of the QOLIBRI-KIDDY. Means (*M*), standard deviations (*SD*), minimum (*Min*), and maximum (*Max*) values were presented for continuous data, and absolute (*n*) and relative frequencies (%) for categorical data.

In most studies, Cronbach’s α values are used to assess the internal consistency of the questionnaire scales. Because of the criticized underestimation of the reliability using Cronbach’s α [52, 53], especially when only few response categories are used and the scale distribution is skewed, we decided to add the reliability information of McDonald’s ω. We required reliability coefficients of at least 0.60 for Cronbach’s α [54, 55] and 0.70 for McDonald’s ω [56–58] based on the sample size and reports of psychometric properties of instruments developed in similar pediatric populations. To assess the impact of each item on the reliability of the corresponding scale, we calculated the effect of omitting an item. We expected that omitting an item would not increase (i.e., improve) the corresponding Cronbach’s α value. It has been hypothesized that in samples of children with cognitive impairment, the internal consistencies of HRQoL scales may be worse [30, 59]. We therefore performed subgroup analyses using the RAVLT learning rate.

Given the sample size, it was not possible to analyze CFA. Thus, we performed scale-wise analyses to assess the goodness of the factor fit and the unidimensionality of the scales. For this purpose, following the suggestion of Revelle & Condon (Revelle & Condon, in Review), we assessed unidimensionality by evaluating the extent to which a single-factor model reproduces the observed correlation matrix (i.e., congeneric fit, *ρ*_*c*_) in relation to the extent to which the mean inter-item correlation aligns with the correlation matrix (i.e., *τ* equivalence). The calculated values of the unidimensionality index (*u*) range from 0 to 1, with values closer to 1 indicating stronger unidimensionality of the scale. Because of the experimental nature of this procedure, no standardized cut-off values have yet been established. We also calculated corrected item-total correlations (CITC) to explore the association between the items and their respective scales. Values of CITC ≥ 0.40 [60] were considered acceptable.

To investigate construct validity, we calculated scale-wise Pearson (*r*) correlations with the PedsQL, which measures generic HRQoL, where the expected overlap would be reflected by a strong positive relationship. The coefficient of determination (R^2^) was additionally used to express the amount of shared variance. Discriminant validity was determined by the relationship between the QOLIBRI-KIDDY scales and the parent-reported measures of anxiety and depression (GAD-7 and PHQ-9, respectively) and the self-reported burden of post-concussion symptoms (PCSI-SR5). Negative associations were expected, reflecting that reduced HRQoL was associated with higher values of anxiety, depression, and post-concussion symptoms, and vice versa.

Finally, we tested the convergent and discriminant validity of the scales with a series of hypotheses reflecting TBI-specific HRQoL, as measured by the QOLIBRI-KIDDY scales, sociodemographic (i.e., sex, age) and clinical known groups (i.e., TBI severity, level of functional recovery, presence of anxiety, depression, post-concussion symptoms, and the presence of cognitive, sensory, and physical problems). Based on previous findings, we expected lower HRQoL in girls [13], those with more severe TBI [15], lower levels of functional recovery [16], higher symptom burden [17, 19, 20], and any cognitive, sensory [14], or physical problems [18] compared to the respective reference groups.

Hypotheses were tested using *t*-tests for independent samples for all other group comparisons. The effect size for group comparisons was determined by Cohen’s d, with values 0.2, 0.5, and 0.80 indicating small, medium, and large effects, respectively [61]. When the number of cases in both groups was n < 20, the analyses were reported as showing at least a trend.

All analyses were performed with R version 4.3.0 [62] applying the packages “psych” [63] and “psychTools” [64] for psychometric analyses and “corrplot” [65] for data visualization. The significance level was set at 5%.

## Results

### Study participants

The mean age of the participants (60% males) was 7.02 ± 0.57 years. The majority of participants attended school (69%). The most common type of TBI they experienced was mild (86%); brain lesions were identified by either CT or MRI in 37.5% of all cases. In 20% of participants, TBI had occurred in the last two years and in nearly half of the sample (46%) more than four years prior to study enrollment. Mothers (76%) were the most common source of parent-reported data. The age of the parents was 40.8 (± 4.34) years and the highest level of family education was mostly university (68%). Descriptive data are shown in Table 1.

**Table 1:** Demographic and clinical characteristics of the participants

Variable	Group	N (%) or M (SD) and Mdn [Min, Max]
**Sociodemographic characteristics (children)**	Total sample	72 (100%)
Age groups	6 years of age	33 (45.8%)
	7 years of age	39 (54.2%)
Age in years	*M* (*SD*)	7.02 (0.57)
	*Mdn* [*Min, Max*]	7.21 [6.00, 7.92]
Sex	Female	29 (40.3%)
	Male	43 (59.7%)
Educational facility	Kindergarten	22 (30.6%)
	School	50 (69.4%)
Type of assessment (face-to-face interview)	Online	47 (65.3%)
	On-site	25 (34.7%)
**Sociodemographic characteristics (parents)**		72 (100%)
Parent interviewed	Mother	55 (76.4%)
	Father	14 (19.4%)
	Missing	3 (4.2%)
Age in years	*M* (*SD*)	40.80 (4.34)
	*Mdn* [*Min, Max*]	41.00 [31.00, 49.00]
	Missing	13 (18.1%)
Highest family educational attainment	Secondary school	9 (12.5%)
	Occupational school	10 (13.9%)
	University	49 (68.1%)
	Missing	4 (5.6%)
Family situation	Single parent	6 (8.3%)
	In relationship	62 (86.1%)
	Missing	4 (5.6%)
Type of data assessment	Paper-pencil (postal or on-site)	72 (100%)
**TBI-related and clinical characteristics**		
TBI severity	Mild Uncomplicated mild^1^ Complicated mild^1^	62 (86.1%) 43 (69.4%) 19 (30.6%)
	Moderate	6 (8.3%)
	Severe	4 (5.6%)
Lesions	No	45 (62.5%)
	Yes	27 (37.5%)
KOSCHI score	4b: upper moderate disability	3 (4.2%)
	5a: good recovery	5 (6.9%)
	5b: full recovery	64 (88.9%)
Time since TBI	< 1 Year	2 (2.8%)
	1–2 Years	12 (16.7%)
	2–4 Years	24 (33.3%)
	≥ 4 Years	33 (45.8%)
	Missing	1 (1.4%)
Health Problems after TBI (proxy-reported)^2^	No	50 (69.4%)
	Yes	22 (30.6%)
RAVLT learning rate	Average/High	36 (50.0%)
	Low (*M -* 1 *SD*)	35 (48.6%)
	Missing	1 (1.4%)
Anxiety (GAD-7; proxy-reported)	No/Minimal (0–4)	51 (70.8%)
	Mild to severe (> 5)	18 (25.0%)
	Missing	3 (4.2%)
Depression (PHQ-9; proxy-reported)	No/Minimal (0–4)	48 (66.7%)
	Mild to severe (> 5)	20 (27.8%)
	Missing	4 (5.6%)
Post-concussion symptoms (PCSI-SR5; self-reported)	Low/Average (*M, M* - 1 *SD*)	56 (77.8%)
	Above average (*M* + 1 *SD*)	10 (13.9%)
	Missing	6 (8.3%)

Note. Due to the inherent limitations of rounding, it is possible that the exact percentage of 100% may not be achieved. *N* = absolute frequencies, *%* = relative frequencies, *M* = mean, *SD* = standard deviation, *Mdn* = median, *Min* = minimum, *Max* = maximum, TBI = traumatic brain injury, RAVLT = Rey Auditory Verbal Learning Test, GAD-7 = Generalized Anxiety Disorder 7-Item Scale, PHQ-9 = Patient Health Questionnaire 9-Item Scale, PCSI-SR5 = Post-Concussion Symptom Inventory. ^1^ Complicated mild = mild TBI but with lesion found in CT and/or MRI; Uncomplicated mild = mild TBI without lesions [66]; ^2^ Parent-reported problems after TBI (sensory, physical, and cognitive problems, other). “Yes” if at least one endorsed

### Item generation and reduction

The evaluation of the item pool resulted in a final version made up of 23 items (see Appendix A, Table A1) forming six scales, corresponding to both the adult QOLIBRI [29] and the QOLIBRI-KID/ADO [30]. Four scales measure satisfaction within the following domains: Cognition (four items), Self (four items), Daily Life and Autonomy (four items), and Social Relationships (three items). Two scales assess the feeling of being bothered by Emotions (three items) and Physical Problems (five items). With Cronbach’s ɑ ranging from 0.49 to 0.72 and McDonald’s ω from 0.57 to 0.78, the reliability coefficients suggest sufficient [55] to good internal consistency of the scales. In the subgroup analyses of the internal consistencies for individual scales and the subgroup with lower learning rates, we found low to good internal consistency values (Cronbach’s α: 0.32–0.75; Appendix A, Table A2).

All scales were considered unidimensional (*u*: 0.70 to 1.00). Most items had CITC ≥ 0.40 with the corresponding scale scores. Five items [“Talking to Others”, “Thinking Speed”, “Self-Esteem”, “Accomplishment”, and “Manage at school”) had CITCs below 0.40. However, they exceeded 0.30, corresponding to a moderate effect according to 61, and this was considered sufficient to demonstrate the items’ association with the respective scales. For details, see Table 2.

**Table 2:** Item and scale characteristics (reliability and validity)

Scale	Item	ɑ	ω	ɑ if item omitted	***τ***	***ρ***_***c***_	u	CITC
Cognition	Concentration (A, KA)	0.49	0.58	**0.42**	**0.89**	**0.78**	**0.70**	**0.44**
	Talking to Others (A, KA)			**0.45**				0.35
	Remembering (A, K/A)			**0.33**				**0.55**
	Thinking Speed (A, K/A)			**0.46**				0.34
Self	Appearance (A, K/A)	0.50	**0.62**	**0.33**	**0.91**	**0.79**	**0.72**	**0.57**
	Self-Esteem (A, K/A)			**0.46**				0.35
	Accomplishment (A, K/A)			**0.49**				0.32
	Feel confident (New)			**0.42**				**0.46**
Daily Life and Autonomy	Daily Independence (A, KA)	0.53	**0.62**	**0.45**	**0.90**	**0.83**	**0.75**	**0.47**
	Manage at school (A, K/A)			**0.54**				0.31
	Social Activities (A, K/A)			**0.39**				**0.54**
	Ability to Move (K/A)			**0.44**				**0.47**
Social Relationships	Family Relationship (A, K/A)	**0.55**	0.57	**0.52**	**1.00**	**0.97**	**0.97**	**0.43**
	Relationship with Friends (A, K/A)			**0.46**				**0.48**
	Demands from Others (K/A)			**0.37**				**0.56**
Emotions	Anger (A, K/A)	**0.72**	**0.72**	**0.63**	**1.00**	**1.00**	**1.00**	**0.62**
	Anxiety (A, K/A)			**0.63**				**0.63**
	Sadness (A, K/A)			**0.62**				**0.64**
Physical Problems	Headaches (A, K/A)*	**0.70**	**0.78**	**0.69**	**0.97**	**0.90**	**0.87**	**0.43**
	Pain (A, K/A)*			**0.68**				**0.47**
	Clumsiness (A, K/A)			**0.61**				**0.66**
	Seeing/Hearing (A, K/A)			**0.59**				**0.71**
	Other Injuries (A, K/A)			**0.67**				**0.49**

Note. * In the adult QOLIBRI version, “Pain” and “Headaches” were assessed in a single question. (A) Items corresponding to items in the adult QOLIBRI version; (K/A) Items corresponding to items in the QOLIBRI-KID/ADO version; *τ* = *τ* equivalence (how well a factor reproduces the covariance matrix under assumption of equal true score variances); ρ_c_ = congeneric fit (how well the observed correlation matrix can be reproduced under assumption of congeneric items (i.e., items with different loadings)); u = unidimensionality measure calculated as a product of *τ* and ρ_c_ with values closer to 1 indicating stronger unidimensionality of the construct; CITC = corrected item-total correlations. Values in **bold** indicate fit indices meeting the following requirements: reliability coefficients > 0.60, “Cronbach’s ɑ if item omitted” lower or equal to the Cronbach’s ɑ of the scale, CITC ≥ 0.40

The descriptive statistics of item and scale scores are presented in Table 3. The mean scores for the “satisfaction” items were mostly higher than four (“quite satisfied”) and moderately to highly skewed to the left (i.e., being more satisfied), as is often seen in other satisfaction studies [67]. The “bothered” items yielded a mean close to three (“moderately bothered”) and mostly showed no skewness, except for the item “Anger”, which was skewed to the right (i.e., being more bothered), and the item “Seeing/hearing” and “Other injuries”, which were left-skewed (i.e., being less bothered). Details concerning the response patterns for each of the items are shown in Appendix A, Table A3.

**Table 3:** Descriptive statistics for the items of the final version of the QOLIBRI-KIDDY

Scale	Item	***M***	***SD***	% Missing	***SK***	% Floor	% Ceiling
Cognition	Concentration (A, KA)	3.77	1.12	1	−0.63	8	57
	Talking to Others (A, KA)	4.34	0.84	1	−0.97	3	81
	Remembering (A, K/A)	4.08	1.02	1	−0.79	7	69
	Thinking Speed (A, K/A)	4.17	0.94	1	−1.25	4	79
Self	Appearance (A, K/A)	4.49	0.78	3	−1.06	0	81
	Self-Esteem (A, K/A)	4.67	0.58	3	−1.54	0	92
	Accomplishment (A, K/A)	4.73	0.56	3	−1.91	0	92
	Feel confident (New)	4.40	0.67	3	−0.64	0	88
Daily Life and Autonomy	Daily Independence (A, KA)	4.38	1.00	0	−1.71	7	85
	Manage at school (A, K/A)	4.46	0.71	0	−0.90	0	88
	Social Activities (A, K/A)	4.60	0.64	0	−1.30	0	92
	Ability to Move (K/A)	4.82	0.45	0	−2.46	0	97
Social Relationships	Family Relationship (A, K/A)	4.25	0.99	0	−1.19	3	75
	Relationship with Friends (A, K/A)	4.46	0.80	0	−1.47	4	89
	Demands from Others (K/A)	3.65	1.33	0	−0.62	21	60
Emotions	Anger (A, K/A)	2.42	1.39	0	0.51	51	19
	Anxiety (A, K/A)	2.92	1.54	0	0.09	40	35
	Sadness (A, K/A)	2.78	1.47	0	0.20	47	35
Physical Problems	Headaches (A, K/A)*	2.82	1.56	0	0.16	47	38
	Pain (A, K/A)*	2.92	1.55	0	0.03	39	38
	Clumsiness (A, K/A)	2.82	1.50	0	0.21	44	33
	Seeing/Hearing (A, K/A)	3.70	1.68	1	−0.74	26	62
	Other Injuries (A, K/A)	3.69	1.64	1	−0.67	26	58

Note. * In the adult QOLIBRI version, “Pain” and “Headaches” were assessed in a single question. (A) Items corresponding to the items of the adult QOLIBRI version; (K/A) Items corresponding to the items of the QOLIBRI-KID/ADO version; *M* = mean, *SD* = standard deviation, *SK* = skewness. Negative values indicate left-skewed distributions

Descriptive statistics for the scales of the QOLIBRI-KIDDY, the PedsQL and the screening instruments GAD-7, PHQ-9, and PCSI-SR5 are presented in (Table 4). The mean “satisfaction” scale scores of the QOLIBRI-KIDDY questionnaire were above 75 (i.e., “quite satisfied”) and left-skewed. The mean “bothered” scale scores were close to 50 (i.e., “moderately bothered”); the Emotions sum scale score was only 42 points and rather symmetrically distributed. The scores of the generic HRQoL instrument PedsQL were left-skewed with means above 75. Overall, the anxiety, depression and post-concussion screening instruments GAD-7, PHQ-9 (around 3) and PCSI-SR5 (<1) indicated minimal symptoms.

**Table 4:** Descriptive statistics of the QOLIBRI-KIDDY, PedsQL, GAD-7, PHQ-9, and PCSI-SR5

Instrument	Scale	***N***	***M***	***SD***	***Md***	***Q1***	***Q3***	***SK***
QOLIBRI-KIDDY	Cognition	71	77.29	15.54	75	68.75	90.62	−0.21
	Self	70	89.29	10.38	93.75	81.25	100	−0.79
	Daily Life and Autonomy	72	89.06	11.43	93.75	81.25	100	−1.46
	Social Relationships	72	78.01	19.19	83.33	66.67	91.67	−0.83
	Emotions	72	42.59	29.29	41.67	16.67	66.67	0.21
	Physical Problems	71	55.14	26.58	55	40	72.5	0.02
PedsQL	Emotional Functioning	72	76.01	20.58	80.00	68.12	90.00	−0.82
	Social Functioning	72	76.84	19.24	80.00	70.00	90.00	−0.77
	School Functioning	72	78.33	16.19	80.00	70.00	90.00	−0.91
	Physical Functioning	72	85.09	12.86	87.5	79.69	93.75	−1.02
	Psychosocial Functioning	72	77.06	14.30	77.08	66.67	86.67	−0.54
	Total Functioning	72	79.85	12.09	82.61	71.20	89.13	−0.59
GAD-7	Total Score	69	3.29	3.00	2	1	5	1.11
PHQ-9	Total Score	68	3.40	3.23	3	1	5	1.74
PCSI-SR5	Total Score	66	0.95	1.26	1	0	1	1.55

Note. *N* = absolute frequencies, *M* = mean, *SD* = standard deviation, *Md* = Median, *Q1* = first quartile (25^th^ percentile of data), *Q3* = third quartile (75^th^ percentile of data), SK = skewness (negative values indicate left-skewed distributions), PedsQL = Pediatric Quality of Life Inventory (self-reported), GAD-7-Item Scale = Generalized Anxiety Disorder 7 (proxy-reported), PHQ-9 = Patient Health Questionnaire 9-Item Scale (proxy-reported), PCSI-SR5 = Post-Concussion Symptom Inventory (self-reported)

**Fig. 1:**
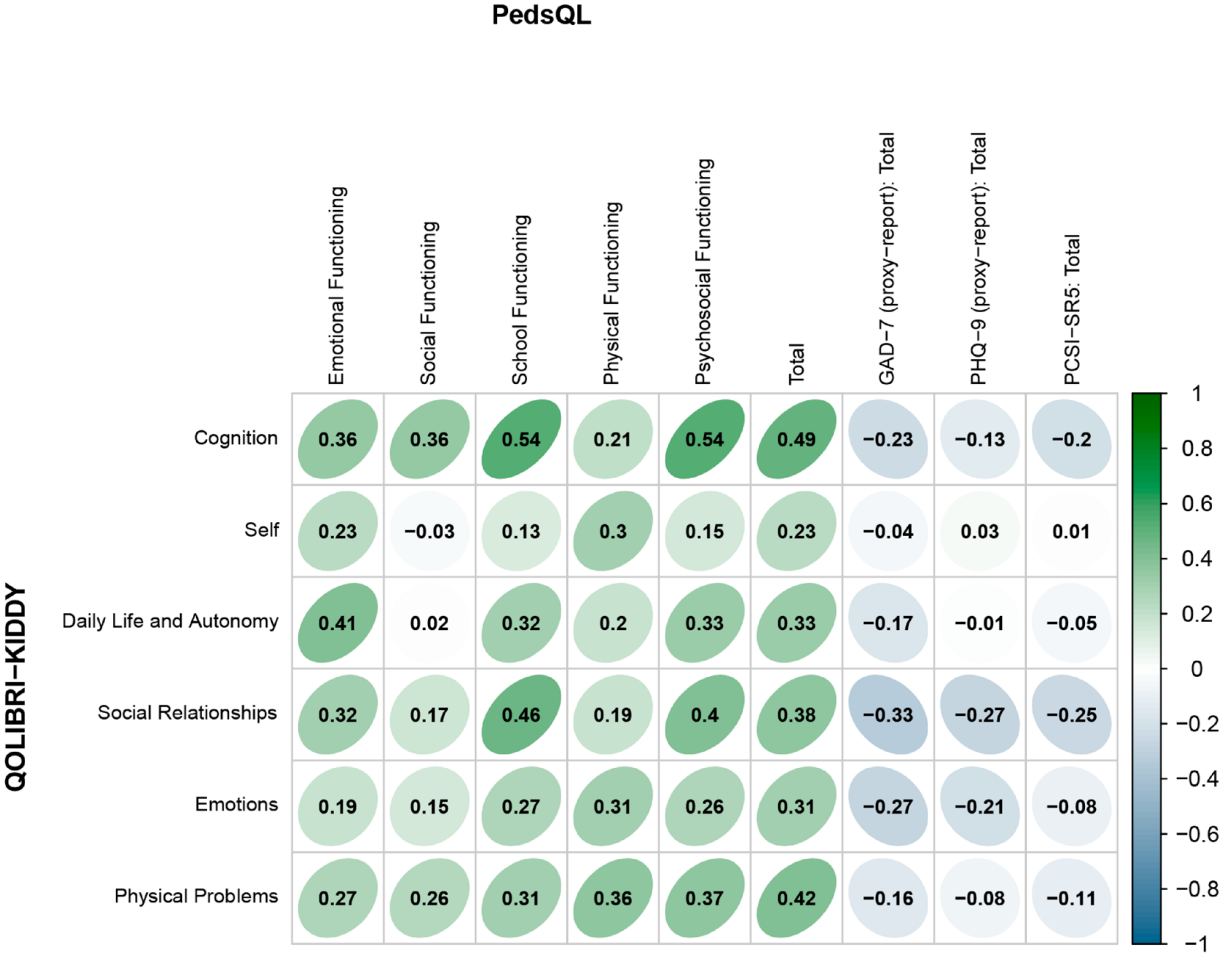
Pearson correlation coefficients between the QOLIBRI-KIDDY scale scores with the PedsQL (self-reported), GAD-7 (proxy-reported), PHQ-9 (proxy-reported), and PCSI-SR5 (self-reported). Note. Green-shaded values indicate positive correlations, blue-shaded values indicate negative correlations, with higher color saturation indicating a stronger relationship

Figure 1 provides an overview of the construct validity analyses using the Pearson correlation coefficients. Correlation coefficients for the QOLIBRI-KIDDY scale scores and the corresponding PedsQL scale and total scores ranged from *r* = 0.17 (R^2^ = 3%; Social Relationships & Social Functioning) and *r* = 0.19 (R^2^ = 4%; Emotions & Emotional Functioning) to *r* = 0.36 (R^2^ = 13%; Physical Problems & Physical Functioning). The amount of variance explained by the QOLIBRI-KIDDY and all PedsQL scales and the total score ranged from 0% to 29%; the highest correlations were found between the Cognition scale of the QOLIBRI and School, Psychosocial Functioning, and Total score of the PedsQL. Overall, the correlations suggest some expected overlap of the assessed generic and TBI-specific HRQoL constructs.

Figure 1 also provides information about the convergent and discriminant validity of the QOLIBRI-KIDDY. The Pearson correlations between the QOLIBRI-KIDDY scale scores and the GAD-7 (*r* = − 0.33 to r = − 0.04), PHQ-9 (*r* = − 0.27 to r = 0.03), and PCSI-SR5 (*r* = − 0.25 to *r* = 0.01) were predominantly negative. Negative values indicated lower TBI-specific HRQoL (lower QOLIBRI-KIDDY scores) as symptoms increase. The total amount of variance explained by the QOLIBRI-KIDDY in terms of symptom burden and vice versa ranged from 0% to 11%, indicating low construct overlap.

Comparative analyses could not be performed for TBI severity, functional recovery, and anxiety burden due to insufficient numbers in at least one group (n < 20). Known-group comparisons concerning sociodemographic and clinical comparisons are shown in Table 5, investigating further aspects of the convergent and discriminant validity of the QOLIBRI-KIDDY questionnaire. Comparing high to no/minimal depression symptom burden, we observed significant differences at the 5% level for the Emotions scale (Δ*M* = − 4. 85, *t*(66) = − 1.72, *p* = 0.045). Regarding the post-concussion symptoms, we detected significant differences for the Cognition (Δ*M* = − 6.49, *t*(63) = − 1.69, *p* = 0.048) and Social Relationships scales (Δ*M* = − 9.93, *t*(64) = − 2.28, *p* = 0.013) of the QOLIBRI-KIDDY.

**Table 5:** Results of the sociodemographic and clinical known-groups comparisons of QOLIBRI-KIDDY scale scores (for subgroups with n > 20)

Groups	Scale	***n1***	***n2***	***M1***	***M2***	***t***	***df***	***p***	***d***	***CI***_***95%***_
**Sex** (Female vs. Male)	Cognition	29	42	74.62	78.55	−1.05	69	0.150	−0.25	[−0.74, 0.23]
	Self	29	41	89.52	88.54	0.39	68	0.649	0.09	[−0.39, 0.58]
	Daily Life and Autonomy	29	43	86.76	90.12	−1.22	70	0.113	−0.29	[−0.78, 0.19]
	Social Relationships	29	43	80.83	75.67	1.12	70	0.867	0.27	[−0.21, 0.75]
	Emotions	29	43	46.76	39.19	1.08	70	0.858	0.26	[−0.22, 0.74]
	Physical Problems	29	42	54.38	55.45	−0.17	69	0.434	−0.04	[−0.52, 0.44]
**Health problems after TBI** (Yes vs. No)	Cognition	21	50	74.38	78.02	−0.90	69	0.186	−0.23	[−0.75, 0.29]
	Self	21	49	88.10	89.31	−0.44	68	0.330	−0.12	[−0.64, 0.41]
	Daily Life and Autonomy	22	50	86.41	89.80	−1.16	70	0.126	−0.30	[−0.81, 0.22]
	Social Relationships	22	50	75.91	78.56	−0.54	70	0.296	−0.14	[−0.65, 0.37]
	Emotions	22	50	37.82	44.18	−0.85	70	0.200	−0.22	[−0.73, 0.3]
	Physical Problems	22	49	47.36	58.45	−1.65	69	0.052	−0.42	[−0.94, 0.1]
Learning rate* (Low learning rate vs. Average/high learning rate)	Cognition	34	36	75.59	77.78	−0.59	68	0.280	−0.14	[−0.62, 0.34]
	Self	33	36	90.36	87.53	1.12	67	0.867	0.27	[−0.21, 0.75]
	Daily Life and Autonomy	35	36	92.09	85.75	2.40	62.05	0.990	0.57	[0.08, 1.05]
	Social Relationships	35	36	78.06	76.83	0.27	69	0.605	0.06	[−0.41, 0.54]
	Emotions	35	36	36.57	46.83	−1.49	69	0.070	−0.35	[−0.83, 0.12]
	Physical Problems	34	36	52.06	56.69	−0.73	68	0.233	−0.18	[−0.65, 0.3]
Depression symptoms** (High vs. No/Minimal symptom burden)	Cognition	20	47	75.60	76.23	−0.15	65	0.439	−0.04	[−0.57, 0.49]
	Self	19	47	-	-	-	-	-	-	-
	Daily Life and Autonomy	20	48	88.75	88.50	0.07	25.73	0.526	0.02	[−0.51, 0.55]
	Social Relationships	20	48	73.40	78.25	−0.95	66	0.174	−0.25	[−0.79, 0.28]
	Emotions	20	48	73.40	78.25	−1.72	66	**0.045**	−0.46	[−1.00, 0.08]
	Physical Problems	20	47	73.40	78.25	−1.40	66	0.083	−0.37	[−0.91, 0.16]
Post-concussion symptoms*** (Average/high vs. Low symptom burden)	Cognition	33	32	73.67	80.16	−1.69	63	**0.048**	−0.42	[−0.92, 0.08]
	Self	33	31	88.64	90.03	−0.53	62	0.298	−0.13	[−0.63, 0.37]
	Daily Life and Autonomy	34	32	86.62	90.94	−1.53	58.74	0.065	−0.37	[−0.87, 0.12]
	Social Relationships	34	32	72.82	82.75	−2.28	64	**0.013**	−0.56	[−1.06, − 0.06]
	Emotions	34	32	41.56	41.81	−0.04	64	0.486	−0.01	[−0.5, 0.48]
	Physical Problems	33	32	52.52	57.47	−0.74	63	0.230	−0.18	[−0.68, 0.31]

Note. *assessed by the Rey Auditory Verbal Learning Test (RAVLT); ** assessed by the Patient Health Questionnaire 9-Item Scale (PHQ-9) ***assessed by the Post-Concussion Symptom Inventory (PCSI-SR5); *n1, n2* = absolute frequencies in the groups following the order in the first column; *M*1, *M*2 = means of the respective QOLIBRI-KIDDY scale scores per group following the order in the first column; *t=t*-test statistic; *df* = degrees of freedom. Decimals are due to the fact that a t-test for heterogeneous variances was calculated for this group; *p=p*-value (**bold**: *p* < 0.05); *d* = Cohen’s d (0.2, 0.5, and 0.80 indicating small, medium, and large effects, respectively); *CI*_*95%*_ = 95% confidence interval

## Discussion

This is the first study to develop and validate a version of the QOLIBRI for very young children, the disease-specific HRQoL QOLIBRI-KIDDY self-report measure for children aged 6–7 years after a TBI. In recent years, subjectively perceived HRQoL has become increasingly important as a PROM to improve the diagnostic and therapeutic process [68]. The current study may support clinical research and practice in this pediatric patient group and complements the recently developed QOLIBRI-KID/ADO for 8–17 year old children and adolescents [30]. Research on HRQoL in children faces several challenges, including the identification of relevant dimensions, the advantages and disadvantages of self-report versus proxy reporting, the comprehensibility of age-appropriate items, and the appropriateness of generic vs. disease-specific instruments [55]. The current QOLIBRI-KIDDY questionnaire attempts to address these challenges. We would like to emphasize that disease-specific instruments are generally more sensitive than generic ones [69], for instance in capturing the specific impact of TBI. The effects of pediatric TBI encompass potential problems in self-awareness [70], autonomy and participation [71–73] as well as in physical [74–77], emotional [77–79], cognitive [77, 80], and social functioning [81] domains. These consequences of pediatric TBI may severely reduce the HRQoL of affected individuals.

Due to the item selection process, based on several expert discussions, focus group interviews, and cognitive debriefings, the items tested in 72 individuals after pediatric TBI were easily understandable for this age group. Following the concept of HRQoL as a subjective report of one’s own subjective well-being [82] and the tendency towards lower agreement when comparing parent and self-reports [11, 83–90], the current study recommends the use of self-reports. In addition, however, the QOLIBRI-KIDDY also offers a parent-report option for patients who cannot self-report anymore. This topic will be addressed in a separate study.

The newly developed QOLIBRI-KIDDY consists of 23 age-appropriate items associated with six domains covering four “satisfaction” (Cognition, Self, Daily Life and Autonomy, Social Relationships) and two “bothered” subscales (Emotions, Physical Problems). The questionnaire is characterized by satisfactory internal consistency. As in multiple pediatric studies, the indices in our study were also partly below the cut-offs expected in general psychometric research with Cronbach’s α > 0.6 [55] and ω > 0.7 [57]. With respect to these standards, the indices were at least acceptable for all subscales. For the two scales measuring how much the children were bothered by Emotions or Physical Problems Cronbach’s α and McDonald’s ω are found to be above 0.7. This implies that the children perceived the items within each subscale to be sufficiently related to reflect a coherent underlying construct. The lower internal consistencies were found only on positively worded scales that measure how satisfied the children are. Interestingly, we identified these lower consistencies only in younger children and not in the 8- to 17-year-olds [30, 91]. This may be attributed to the limited number of items included in the instrument, which was designed to be brief and practical for use in very young children. We retained only those items that contributed to the internal consistency of the subscales as measured by Cronbach’s α and McDonald’s ω and whose content was as similar as possible to that of the QOLIBRI-KID/ADO for 8–17 years and the adult version of the QOLIBRI. The rationale behind this was to provide an instrument suitable for measuring TBI-specific HRQoL across the lifespan. Furthermore, a response bias towards either negatively or positively worded items, with children consistently responding differently to both, could also partially explain this phenomenon [92]. We expect that the younger the children are, the more pronounced this effect would be. This assumption needs further investigation in future studies. It may also be difficult for some young children to report reliably on such questions because they are responding on the basis of situational and unstable impressions impacting their self-concept [93] or unstable attribution styles/control beliefs [94]. However, based on the findings from our focus group interviews with children aged 5 to 17 [40] and the results of the current pilot study, we believe that children at least from the age of 5 years on can respond reliably with respect to their HRQoL, albeit with higher response variability due to the particularities of this age group. Furthermore, validation of the QOLIBRI-KIDDY questionnaire in more individuals after moderate and severe TBI with more severe impairments and those with persistent symptoms years after TBI may provide further insights into this issue of lower internal consistency.

Due to the small sample size, a CFA could not be performed to examine the six-factor scale structure. We assessed the goodness of the factor fit and the unidimensionality of the scales using scale-wise analyses. These results provide a first indication of unidimensionality, although there are no conventional cut-offs to apply for further interpretation. The items appear to adequately reflect the underlying construct, as indicated by both the factor fit values and their product. Future studies should focus on the factorial validity of the QOLIBRI-KIDDY to verify whether the theoretical six-factor structure adapted from the adult QOLIBRI adequately reflects TBI-specific HRQoL in young children.

The results of the study showed that the majority of the children were quite satisfied (values > 75) and felt moderately bothered (values close to 50). The tendency to report higher levels of satisfaction may be explained by the participants’ characteristics, as the sample only contained few individuals after moderate or severe TBI [11, 95], with disabilities or chronic conditions [96], or with cognitive impairments [72, 97–100]. To date, only generic HRQoL instruments assessed after TBI have been published and no HRQoL studies with young children (i.e., under the age of eight) after TBI using disease-specific TBI instruments [11]. Compared with generic instruments [36, 55], the QOLIBRI-KIDDY instrument has the great advantage of assessing specific consequences of TBI, such as headaches or other injuries caused by the accident, and emotional or cognitive problems, such as memory or processing speed. Interestingly, in contrast to the “satisfaction” scales, the Emotions and Physical Problems scales showed that, on average, the children were moderately bothered by TBI-related problems and had significantly lower HRQoL scores compared with studies assessing generic HRQoL. This could be explained by the so-called cognitive-developmental phenomenon [101] at this young age, whereby the negative item bias results from the inability of pre-adolescent children to respond appropriately to negative items on rating scales. During the interviews, it was noticeable that the young children answered questions involving negative connotations (“How much does it bother you?”) much more decisively than positive questions (“How satisfied are you?”), i.e., they often answered that they were definitely “very” or “quite” bothered by something. The fact that a response bias may exist for negatively or positively worded items [92] and that the interpretation of negatively worded items may differ from that of positively worded items [101] might therefore be part of the explanation. We recommend investigating this possible response bias in future studies.

Validity analyses showed that the construct of the specific instrument revealed some expected overlaps with the generic HRQoL measure PedsQL. The QOLIBRI-KIDDY physical, social, and emotional scales showed small to medium positive correlations with the corresponding scales of the PedsQL. Similar associations of the QOLIBRI-KID/ADO were found to be even stronger for older children and adolescents [30] as well as between the adult TBI-specific QOLIBRI measure [26, 29, 102] and the physical and mental composite score of the SF-36 [103]. We therefore conclude that our measure is assessing TBI-specific HRQoL aspects which are not covered by generic instruments. This again suggests using measures that are designed for specific health conditions, such as QOLIBRI-KIDDY.

The distribution of sex and severity matches the epidemiological patterns found in Germany [104] with more boys affected (60%) than girls (40.0%) and with the majority of the children (86%) suffering a mild TBI, whereby 30% of them had a complicated mild TBI with lesions identified in imaging. We found no association between sex and HRQoL, and due to the insufficient numbers, we were not able to analyze the potential effect of TBI severity on HRQoL. However, we found moderate negative correlations between HRQoL, particularly the Social Relationships and Emotions scales, and depression and anxiety. According to previous research, between 11% and 45% of children after pediatric TBI are prone to mental health problems [78]. In our study, 25% to 28% of children showed symptoms of anxiety and depression even up to seven years after TBI. There was a significant association between lower HRQoL on the Emotions scale and symptoms of depression. Similar results have been found for lower HRQoL in children with long-term illnesses and mental health problems [14]. Following pediatric TBI, early identification and treatment of emotional problems in children and adolescents may help to improve and accelerate their recovery and HRQoL. In the current study, participants who reported more post-concussion symptoms (14%) across a wide range of emotional, cognitive, physical, and sleep characteristics displayed significantly lower scale scores on the QOLIBRI-KIDDY Cognition and Social Relationships scales, indicating a lower HRQoL than those who had fewer symptoms. The prevalence of post-concussion symptoms in the current study is consistent with previous research which reports a prevalence of 14% to 29% following pediatric TBI [105]. Although symptoms can persist for years after injury, post-concussion symptoms are most evident in the first year following TBI, especially after a mild TBI [106]. The results of our study support the importance of assessing post-concussive symptoms as early as possible and considering potential symptoms in therapeutic settings.

### Strengths and limitations of the study

The main strength of the present study is that it fills the gap of the need for a TBI-specific HRQoL instrument for young children aged 6 to 7 years. It is important to acknowledge the limitations of this study. Less than 10% of children with TBI who were invited to participate in the study actually enrolled. This decision was made by their parents and was entirely voluntary. Due to privacy restrictions, it was not possible to analyze the differences between participants and non-participants. But a self-selection bias [107] may potentially explain the high drop-out rate observed. The reasons for non-response remain speculative. Potential factors that may have contributed to this include relocation, unwillingness to participate, parental worries, such as the belief that their children were too healthy to participate, or concerns about re-traumatizing the children.

In addition, as in many observational studies concerning TBI [11], most of our study`s participants had experienced a mild TBI with good recovery trajectories. This reflects the epidemiology of pediatric TBI, with 91 to 97% of individuals in Germany having undergone a mild TBI [104]. However, we expect that even young children after more severe TBI and limited functional recovery will comprehend the questionnaire`s items. This expectation is especially based on the equally structured and repetitive nature of our question format. Despite the more complex formulation of the self-reported items, the PedsQL questionnaire has been successfully used in clinical cohorts, including more than 25,000 children aged 5 to 18 years [33], or children after moderate and severe TBI [15].

The small sample size limited our analyses and the generalizability of the results. Especially when testing hypotheses on known groups, small effects could not be detected.

Due to the small sample size, a CFA could not be performed to examine the six-factor scale structure. Although the scale-specific characteristics do provide an indication of the unidimensionality of the six theoretical scales, they cannot serve as a substitute for CFA in terms of investigating the factorial structure. In line with this finding, a total score should only be used with caution for comparing and interpreting HRQoL in young children until further evidence is available.

### Outlook

The validation of the questionnaire requires further steps and analyses, some of which will be performed in the final validation study. For example, when testing hypotheses on known groups, small effects could not be detected. In particular, it would be important to further investigate how lower HRQoL might be associated with female sex, higher severity, and lower recovery status. With a larger sample, further comparisons of HRQoL in different age groups (i.e., 6–7, 8–12, and 13–17 years) may supply valuable information on the validity of the QOLIBRI instruments. This should be integrated in the final validation study, which is currently ongoing. In addition, due to the small sample size in the current study, particular attention should be paid to analyses of test-retest reliability and factorial validity.

We recommend that future studies recruit a larger sample, including more individuals after moderate and severe TBI, more recent TBI, and lower recovery grades, to increase their statistical power, validity, and generalizability. In addition, as with the adult version, further linguistic translations and validations of the QOLIBRI-KIDDY should be undertaken in other language contexts to enable TBI-specific HRQoL to be assessed multi-nationally. This will provide clinicians and researchers with a tool for young TBI survivors to further compare the impact of TBI and different types of therapy and care in different cultural and linguistic settings.

## Conclusions

The QOLIBRI-KIDDY questionnaire is the first self-report instrument specifically designed to assess HRQoL in young children aged 6 to 7 years after pediatric TBI. The development of the QOLIBRI-KIDDY questionnaire represents a significant advance in the field of pediatric TBI and its impact on the HRQoL in this age group. Despite the limitations described, it demonstrates promising psychometric properties, ensuring its feasibility and reliability across six HRQoL dimensions and providing a solid foundation for further research. It effectively reflects the theoretically postulated connections between disease-specific HRQoL and related generic constructs [69] providing evidence of its construct validity.

Pediatric TBI can have a wide range of physical, psychological, cognitive, emotional, and psychosocial consequences in younger and older children and adolescents [98]. These symptoms are often initially subtle and manifest gradually, until they become apparent at a later age [78]. Evaluating TBI-specific HRQoL facilitates the identification of such impairments at an early stage, before they become more severe or chronic. Symptoms such as concentration disorders, headaches, emotional problems, or social difficulties that are left untreated or recognized too late may otherwise persist and worsen over time. We recommend multidimensional assessments at the earliest possible stage after TBI, particularly integrating the measurement of TBI-specific HRQoL and post-concussion symptoms. Based on these evaluations, interventions could be personalized taking into account age, gender, and mental health. This approach may potentially mitigate or prevent the chronification of adverse short- and long-term effects of TBI on HRQoL. Training of clinicians concerning the evaluation of HRQoL in children after TBI could facilitate the use of the QOLIBRI-KIDDY instrument in clinical practice. We conclude that the QOLIBRI-KIDDY should be used in research studies and should be incorporated into medical practice to develop rehabilitation guidelines for organizing and improving interventions and post-TBI outcomes.

## Appendix A

**Table A1:** Final item list of the QOLIBRI-KIDDY

Scale	Item description	German items	English items*
Part I		Wenn Du an jetzt und die letzte Woche denkst, wie zufrieden bist Du damit,….	Thinking about now and the past week, how satisfied are you with….
Cognition	Concentration	wie Du Dich im Kindergarten/in der Schule konzentrieren kannst?	how you are able to concentrate at kindergarten/school?
	Talking to Others	wie Du mit anderen reden kannst?	how you can talk with others?
	Remembering	wie Du Dich an etwas erinnern kannst (zum Beispiel, was Du im Kindergarten/Unterricht gemacht hast)?	how you are able to remember something (for example, what you did in kindergarten/class)?
	Thinking Speed	wie schnell Du denken kannst (zum Beispiel, wie lange Du nachdenken musst, um eine Antwort zu geben)?	how fast you are able to think (for example, how long you have to think to give an answer)?
Self	Appearance	wie Du etwas hinbekommst?	with how you manage to do something?
	Self-Esteem	wie Du aussiehst?	with the way you look?
	Accom-plishment	mit Dir, so wie Du bist?	with yourself as you are?
	Feel confident	was Du Dir zutraust?	with what you feel confident about doing?
Daily Life and Autonomy	Daily Independence	ohne Hilfe Dinge zu tun, die Du jeden Tag machst (zum Beispiel Dich anzuziehen)?	being able to do things you do every day without help (for example, getting dressed)?
	Manage at school	wie Du im Kindergarten/in der Schule zurechtkommst?	how you manage at school?
	Social Activities	wie Du bei Deinen Freunden mitspielen kannst (zum Beispiel im Kindergarten/in der Schule, bei Geburtstagsfeiern)?	how you are able to play/do things with your friends (for example, at kindergarten, school, or birthday parties)?
	Ability to Move	wie Du Dich bewegen kannst (zum Beispiel zu gehen, zu rennen, mit dem Rollstuhl zu fahren)?	how you are able to move around (for example, walk, run, use a wheelchair)?
Social Relation-ships	Family Relationship	wie Du Dich mit Deiner Familie verträgst?	how you get along with your family?
	Relationship with Friends	wie Du Dich mit Deinen Freunden verträgst?	how you get along with your friends?
	Demands from Others	was andere von Dir verlangen?	what others demand of you?
**Part II**		**Wenn Du an jetzt und die letzte Woche denkst, wie sehr stört es Dich/stören Dich…**	**Thinking about now and the past week, how much are you bothered by…**
Emotions	Anxiety	ängstlich zu sein?	feeling anxious?
	Sadness	traurig zu sein?	feeling sad?
	Anger	wütend zu werden?	getting angry?
Physical Problems	Clumsiness	wenn Du ungeschickt bist (zum Beispiel, wenn Du stolperst, Dir etwas runterfällt)?	being clumsy (for example, when you trip or drop something)?
	Pain	andere Verletzungen, die Du gleichzeitig bei Deinem Unfall/Deiner Hirnverletzung abbekommen hast?	other injuries you got at the same time as your accident/brain injury?
	Headaches	Kopfschmerzen?	headaches?
	Other Injuries	andere Schmerzen (außer Kopfschmerzen)?	other pain (apart from headaches)?
	Seeing/Hearing	Probleme beim Sehen/Hören?	problems seeing/hearing?

Note. * The English items are based on the developed German items according to the linguistic translation process with two forward and one backward translation, and the final harmonization (ensuring comparability with the adult questionnaire as well as the QOLIBRI-KID/ADO questionnaire), except for the cognitive debriefings with children (in progress)

**Table A2:** Cronbach’s α of total and scale scores of the QOLIBRI-KIDDY for individuals with higher and lower learning rates

QOLIBRI-KIDDY scales	N_High_	**N**_Low_	α_High_	α _Low_
Cognition	35	36	0.60	0.33
Self	35	36	0.54	0.48
Daily Life and Autonomy	35	36	0.61	0.32
Social Relationships	35	36	0.52	0.57
Emotions	35	36	0.78	0.62
Physical Problems	35	36	0.73	0.65
Total Score	35	36	0.84	0.75

Note. McDonald’s ω could not be estimated in this subgroup analysis

**Table A3:** Item endorsements and response patterns

			Not at all		Slightly		Moderately		Quite		Very		Missing	
QOLIBRI-KIDDY scales	Items	***N***	***n***	%	***n***	%	***n***	%	***n***	%	***n***	%	***n***	%
Cognition	Concentration (A, K/A)	71	4	6	2	3	24	33	17	24	24	33	1	1
	Talking to Others (A, K/A)	71	0	0	2	3	11	15	19	26	39	54	1	1
	Remembering (A, K/A)	71	1	1	4	6	16	22	17	24	33	46	1	1
	Thinking Speed (A, K/A)	71	2	3	1	1	11	15	26	36	31	43	1	1
Self	Appearance (A, K/A)	70	0	0	0	0	12	17	12	17	46	64	2	3
	Self-Esteem(A, K/A)	70	0	0	0	0	4	6	15	21	51	71	2	3
	Accomplishment (A, K/A)	70	0	0	0	0	4	6	11	15	55	76	2	3
	Feel confident (New)	70	0	0	0	0	7	10	28	39	35	49	2	3
Daily Life and Autonomy	Daily Independence (A, K/A)	72	2	3	3	4	6	8	16	22	45	63	0	0
	Manage at school (A, K/A)	72	0	0	0	0	9	13	21	29	42	58	0	0
	Social Activities (A, K/A)	72	0	0	0	0	6	8	17	24	49	68	0	0
	Ability to Move (K/A)	72	0	0	0	0	2	3	9	13	61	85	0	0
Social Relationships	Family Relationship(A, K/A)	72	2	3	0	0	16	22	14	19	40	56	0	0
	Relationship with Friends (A, K/A)	72	0	0	3	4	5	7	20	28	44	61	0	0
	Demands from Others (K/A)	72	7	10	8	11	14	19	17	24	26	36	0	0
Emotions	Anger (A, K/A)	72	28	39	9	13	21	29	5	7	9	13	0	0
	Anxiety (A, K/A)	72	20	28	9	13	18	25	7	10	18	25	0	0
	Sadness (A, K/A)	72	20	28	14	19	13	18	12	17	13	18	0	0
Physical Problems	Headaches (A, K/A)*	72	22	31	12	17	11	15	11	15	16	22	0	0
	Pain (A, K/A)*	72	22	31	6	8	17	24	10	14	17	24	0	0
	Clumsiness (A, K/A)	72	20	28	12	17	17	24	7	10	16	22	0	0
	Seeing/Hearing (A, K/A)	71	16	22	3	4	7	10	5	7	40	56	1	1
	Other Injuries (A, K/A)	71	14	19	5	7	10	14	2	3	40	56	1	1

Note. Due to the inherent limitations of rounding, it is possible that the exact percentage of 100% may not be achieved. * In the adult QOLIBRI version, “Pain” and “Headaches” were assessed in a single question. (A) Items corresponding to the items of the adult QOLIBRI version; (K/A) Items corresponding to the items of the QOLIBRI-KID/ADO version; *N* = absolute frequencies per item, *n* = absolute frequencies per response category, *%* = relative frequencies

## Data Availability

The datasets used and analyzed during the current study are available from the corresponding author on reasonable request.
